# Disparities and determinants of maternal health services utilization among women in poverty-stricken rural areas of China: a cross-sectional study

**DOI:** 10.1186/s12884-023-05434-7

**Published:** 2023-02-14

**Authors:** Yuxuan Yang, Min Yu

**Affiliations:** Department of Health Service Support Research, Academy of Military Medical Sciences, #27 Taiping Road, Haidian District, Beijing, 100850 China

**Keywords:** Maternal health services utilization, Andersen’s Behavioral Model of Health Services Use, Antenatal care, Institutional delivery, Postnatal care

## Abstract

**Background:**

Reducing maternal mortality ratio (MMR) has been a worldwide public health challenge for a long time. Utilization of maternal health services including antenatal care (ANC), institutional delivery (ID), and postnatal care (PNC) is vital to prevent maternal mortality. China has made significant improvements in maternal health during the past 30 years, however, disparities in maternal health service utilization still exist among regions and the western rural areas had the lowest utilization rate. This study aims to assess the inequality and determinants of maternal health service utilization in western poverty-stricken rural areas based on Anderson’s Behavioral Model of Health Service Use and provide evidence-based suggestions to improve equity and coverage of maternal service utilization in China.

**Methods:**

A cross-sectional study was conducted in Gansu and Yunnan Province, Western China using primary data (*n* = 996) collected by the research team. A multistage, judgment, quota sampling procedure was employed to select the participants of the survey. Trained local health staff formed an interview team to help respondents answer a structured, pre-tested questionnaire designed based on Anderson’s model. Data collected through interviews were used for descriptive analysis, range analysis, and univariate and multivariate binary logistic analysis to identify influencing factors of 5 + ANC, 8 + ANC, ID, and 2 + PNC utilization.

**Results:**

Place of residence, age, education level, annual income, and health education during ANC were influencing factors of 5 + ANC; place of residence, education level, per capita household income, conditional cash transfer (CCT) participation, and distance to health facilities were influencing factors of 8 + ANC; place of residence, education level, and availability of financial incentive programs were influencing factors of ID; number of children, health education during ANC, CCT projects participation, and self-rated health status were influencing factors of 2 + PNC.

**Conclusions:**

Inequalities in maternal service utilization exist between Yunnan and Gansu provinces. This study shows a strong association between both predisposing and enabling factors and maternal services utilization. Predisposing factors such as place of residence, education level, and number of children, enabling factors such as CCT participation, annual income, health education during ANC, and distance to health facilities along with need factor self-rated health status all contribute to maternal services utilization.

We conclude that many factors influence maternal service utilization and interventions targeted at various levels should be considered. Therefore, we suggest more health resources should be invested in underutilized areas, financial incentive projects targeting pregnant women should be implemented, and health education should be provided to improve women’s health literacy.

## Background

### Global context

Every 2 min in 2017, a woman died of causes relating to pregnancy or childbirth [1, 2]. Reducing the maternal mortality rate has been a worldwide public health challenge for a long time and was written in the Sustainable Development Goals (SDGs) in 2015 [3]. SDG target 3.1 aimed to reduce MMR to less than 70 per 100,000 live births by 2030, which is hard to achieve without quality antenatal care (ANC), institutional delivery (ID), and postnatal care (PNC). Studies have proved that adequate utilization of maternal health services is vital to prevent complications during pregnancy and childbirth [4]. ANC visits help detect the risks and complications during pregnancy and promote health education, nutritional consumption, and institutional delivery. The risk of a woman who doesn’t receive any ANC visit dying from pregnancy-related causes is 61 times higher than a woman who receives at least 8 ANC visits [5]. Institutional delivery with skilled birth attendance and timely PNC is also advocated by the WHO to manage emergencies and protect women’s and newborns’ lives. Compared with 2000, more women in 2021 had access to ANC services, where 88.31% of women in the world received at least 1 ANC visit during their pregnancy [6]. However, only 66.29% of women in the world received over 4 ANC visits during their pregnancy, only 79.19% of women were able to deliver their babies in health facilities, and only 68.60% of women received PNC in 2021 [6].

According to the WHO, about 99% of maternal mortality happened in developing countries [2]. The risk of a woman dying from pregnancy and childbirth-related causes is 33 times higher in a developing country than in a developed country [7]. Maternal mortality is more likely to happen in rural and poverty-stricken areas. Disparities in not only maternal mortality rate, but also maternal service utilization existed between developed countries and developing countries, and between urban and rural areas, which hinders the improvement of overall maternal health [8, 9]. In developed countries like the US and UK, over 90% of the women received at least 4 ANC visits as early as 2010, however, countries like Afghanistan and Bangladesh still struggled under 50% in 2021 [6]. Studies found that the “resource-rich” population (urban and rich residents) used significantly more maternal care services compared with the “resource-poor” population (rural and poor residents) [9–11].

### China context

Following the formulation of Law of the People's Republic of China on Maternal and Infant Health Care in 1995, improving maternal and child health has been a public health priority. In 2009, maternal health services were included in the Basic Public Health Service (BPHS) project, aiming to reduce service utilization gaps between resource-rich and resource-poor populations, and among regions. With BPHS, all pregnant women can receive 5 free ANC visits and 2 free PNC visits [12]. By collaborating with UNICEF, China was able to launch a conditional cash transfer (CCT) project on maternal services for poor women living in remote areas from 2011 to 2020 [13]. Women participating in CCT got cash rewards after receiving ANC and PNC visits and delivering in health facilities. As a result of all these projects and efforts, China made big progress in reducing maternal mortality, improving maternal health, and promoting maternal health service utilization. The maternal mortality rate dropped from 88.8 per 100,000 live births in 1992 to 16.9 per 100,000 live births in 2020 [14]. Compared with 1992, in 2020, the proportion of women receiving at least 1 ANC visit increased from 69.7% to 97.4%, the proportion of women delivering in a health facility increased from 57.7% to 99.9%, and the proportion of women receiving PNC visit increased from 69.7% to 95.5% [15]. However, inequality and underutilization of health services still happen in some regions. In 2018, the 5 + ANC rate (percentage of women receiving at least 5 ANC visits) in western urban areas was only 74.7% [15]. Studies in China (2020,2016) revealed that the probability of a woman living in an urban area receiving at least 5 ANC visits was 22.3% higher than that who live in a rural area [16], and they are 1.34 times more likely to receive PNC visits [17]. Another study in 2022 found that the higher the annual personal income, the higher the institutional delivery rate [18]. Most studies on maternal health in China focus on quantifying the improvements in maternal health over time, evaluating the health outcome of different health intervention policies, and accessing disparities in service utilization among regions [19–25]. Only a few studies try to find out the associated factors of maternal service utilization [26–28].

### Conceptual framework

**Fig. 1:**
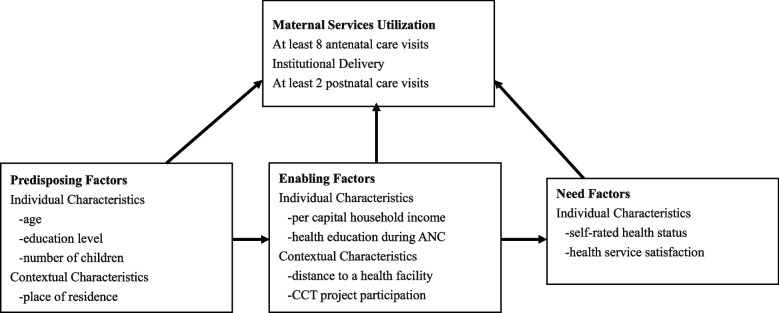
Behavioral model of health service use including predisposing, enabling, and need factors

Behavioral Model of Health Services Use developed by R.M. Andersen has been widely used by researchers to identify determinants of health service utilization. In the original model, Andersen defined three primary determinants of health services use: predisposing factors including socio-demographic characteristics of the population, enabling resources such as income and access to care, and perceived need factors [29]. The model was later updated to an advanced version with distinguishes between contextual factors and individual factors [30]. For a better understanding, the model related to the present study is illustrated in Fig. 1.

Using Anderson’s model has the advantage of considering a wide range of characteristics that may influence health service use. To the best of our knowledge, only a few studies applied this framework to maternal services use [31–34]. Furthermore, studies using Anderson’s model to identify influencing factors of maternal health service utilization in China were lacking. To address this research gap, the present study was conducted to explore different predisposing, enabling, and need factors associated with maternal health service utilization in poverty-stricken rural areas in China to provide evidence-based information that helps provide ideas to improve equity and coverage on maternal service utilization.

## Methods

### Data source

This study was conducted in Gansu and Yunnan Province in China from September 2021 to March 2022. Both provinces are located in the west of China, where women in rural areas had a relatively low maternal health service utilization rate in 2018 [15]. Both provinces were on the list of the top 5 provinces in China with the largest number of impoverished counties [35]. At the same time, 5 counties from each province were chosen to participate in UNICEF’s pilot CCT project from 2011 to 2020.

### Study design and sampling

A cross-sectional study was conducted using a multistage, judgment, quota sampling procedure to select the participants of the study. In the first stage, two counties were selected in each province based on their poverty level (one country-level and one provincial-level county). In the second stage, two towns (one enrolled in CCT project and one didn’t) were selected in each county randomly. In the third stage, five villages were selected in each town based on country doctors’ recommendations. In each village, 30 women who delivered babies from 2011 to 2020 were selected based on convenience sampling strategy to answer surveys. In total, 996 women conducted surveys with a response rate of 83%.

### Data collection tools and procedures

Trained local health staff formed an interview team to help respondents answer a structured, pre-tested questionnaire designed based on Anderson’s model of health service utilization to collect information on respondents’ socio-demographic characteristics, economic conditions, and maternal health service utilization status. Data collectors were given a one-day training about the aim of the study and went through the questionnaire question by question before the actual work. A half-day survey activity were held in each village under research team’s supervision to avoid bias. Invitation messages were sent to all women that delivered babies from 2011 to 2020 to join the activity, the first 30 women that came to the activity were selected to participate in the survey.

### Outcome variables

Both WHO and National Health Commission (NHC) provide guidelines for a list of maternal services that need to be consumed during a woman’s pregnancy period. The present study used 4 variables on the utilization of maternal services for the analysis described as follows:

- 5 + ANC Visits: The Chinese government required at least 5 antenatal visits for a pregnant woman during her pregnancy. The variable was converted into a binary variable (0 = less than 8 ANC visits, 1 = at least 8 ANC visits).
- 8 + ANC Visits: WHO suggested at least 8 antenatal visits for a pregnant woman to minimize pregnancy-related health risks [36]. The variable was converted into a binary variable (0 = less than 8 ANC visits, 1 = at least 8 ANC visits).
- Institutional Delivery (ID): Women are recommended to deliver their babies in a qualified health institution under the supervision of skilled birth attendances. The variable was converted into a binary variable (0 = home delivery, 1 = birth delivery in a qualified health institution).
- 2 + PNC: BPHS provided 2 post-natal checkups free to women within 42 days after live birth and the first checkup was recommended to be done within 24 h which was the same as the WHO recommendation [12, 36]. The variable was converted into a binary variable (0 = less than 2 PNC checkups, 1 = at least 2 PNC checkups).

### Independent variables

Altogether 10 variables were selected and divided into predisposing, enabling, and need factors.

#### Predisposing factors

Place of residence (Gansu, Yunnan); age (≤ 25, 26–30, > 30); education level (primary school or lower, junior high school, senior high school or higher); number of children (1, 2, 3 or more).

#### Enabling factors

Per capita household income (≤ 4000 yuan, > 4000 yuan), 4000 yuan is the extreme poverty line set by the Chinese government in 2020 [37]; distance to health facility (< 2, 2–4, 4–6, > 6 km); health education during ANC visits (yes, no); CCT participation (yes, no).

#### Need factors

Perceived health needs (low, medium, high); health service satisfaction (low, medium, high).

### Data processing and analysis

Computer software SPSS26.0 was used to analyze data. Descriptive analysis was performed at first to describe the frequency and percentage of independent variables. Range method was used to assess the disparities of different maternal service utilizations based on respondents’ socioeconomic status. Then univariable logistic regression was used to find out the relationship between study variables and outcome variables. Multivariable logistic regression analysis was performed afterward to explore determinants of 5 + ANC visits, 8 + ANC visits, ID, and PNC utilizations. All independent variables with $p$ values smaller than 0.2 in univariable analysis were included in multivariable models. Odds ratios (OR) with 95% confidence intervals (CIs) were computed.

## Results

### Characteristics of the respondents

Table 1 presents the characteristics of respondents based on predisposing, enabling, and need factors. For predisposing factors, nearly 41% of the respondents lived in Gansu province and 59% lived in Yunnan province. About 23% of the respondents were under 26 years old, 35% were aged 26–30, and 42% were over 30 years old. Only 19% finished senior high school education or higher. Over 55% of respondents had two children. As for enabling factors, about 57% of respondents had a per capita household income of over 4000 yuan. Over 45% of respondents lived over 6 km away from a health facility. About 92% of respondents were health educated during ANC visits and about 52% of respondents received cash rewards sponsored by CCT after completing ANC, ID and PNC visits. As for need factors, over 64% had high self-rated scores, and over 84% were highly satisfied with the health services they received.

**Table 1:** Respondent’s characteristics divided into predisposing, enabling, and need factors

**Background Characteristics**	Case(n)	Percent
**Predisposing Factors**		
Place of residence		
Gansu	406	40.80
Yunnan	590	59.20
Age		
≤ 25	227	22.79
26–30	352	35.34
> 30	417	41.87
Education Level		
Primary school or below	382	38.35
Junior high school	423	42.47
Senior high school or above	191	19.18
Number of children		
1	183	18.37
2	553	55.52
≥ 3	260	26.10
**Enabling factors**		
Per capita household income (yuan)		
≤ 4000	430	43.17
> 4000	566	56.83
Distance to health facility (km)		
< 2	127	12.75
2–4	147	14.76
4–6	265	26.61
> 6	457	45.88
Health education during ANC		
No	80	8.03
Yes	916	91.97
CCT participation		
No	476	47.79
Yes	520	52.21
**Need Factors**		
Self-rated health status		
Low	74	7.43
Medium	283	28.41
High	639	64.16
Health service satisfaction		
Low	26	2.61
Medium	132	13.25
High	838	84.14
**Total**	996	100

### Respondent’s characteristics and maternal health service utilization

Respondent’s characteristics and maternal health service utilization are presented in Table 2. As for predisposing factors, respondents who lived in Yunnan province had higher utilization of maternal health services besides ID. Respondents aged 26–30 reported higher utilization in 5 + ANC, ID and 2 + PNC. Education level was positively associated with health service utilization. Number of children was negatively associated with health service utilization. As for enabling factors, respondents with over 4000 yuan annual income or received health education reported higher utilization rates. CCT participation was positively associated with 8 + ANC, ID and 2 + PNC. Regarding need factors, respondents with high satisfaction with health services reported higher utilization in 5 + ANC, 8 + ANC and 2 + PNC. More details are presented in the table.

**Table 2:** Percentage of respondents who utilized maternal health services based on background characteristics

Background Characteristics	5 + ANC (n)	8 + ANC (n)	ID (n)	2 + PNC (n)
**Predisposing Factors**				
Place of residence				
Gansu	70.94(288)	15.00(61)	95.07(386)	90.39(367)
Yunnan	95.76(565)	46.30(273)	92.03(543)	95.93(566)
Age				
≤ 25	85.90(195)	31.28(71)	92.07(209)	93.83(213)
26–30	90.34(318)	33.24(117)	95.74(337)	94.32(332)
> 30	81.53(340)	35.01(146)	91.85(383)	93.05(388)
Education				
Primary school or below	73.82(282)	19.60(75)	90.58(346)	89.53(342)
Junior high school	91.96(389)	33.10(140)	93.62(396)	95.27(403)
Senior high school or above	95.29(182)	62.30(119)	97.91(187)	98.43(188)
Number of children				
1	91.80(168)	42.62(78)	92.35(169)	98.36(180)
2	87.88(486)	37.97(210)	94.94(525)	94.39(522)
≥ 3	76.54(199)	17.69(46)	90.38(235)	88.85(231)
**Enabling factors**				
Per capita household income (yuan)				
≤ 4000	79.71(220)	27.21(117)	91.63(394)	91.86(395)
> 4000	87.92(633)	38.34(217)	94.52(535)	95.05(538)
Distance to health facility (km)				
< 2	85.83(109)	15.75(20)	96.06(122)	94.49(120)
2–4	82.99(122)	30.61(45)	93.20(137)	89.80(132)
4–6	80.75(214)	20.00(53)	92.83(246)	88.30(234)
> 6	89.28(408)	47.26(216)	92.79(424)	97.81(447)
Health education during ANC				
No	57.50(46)	22.50(18)	91.25(73)	68.75(55)
Yes	88.10(807)	34.50(316)	93.45(856)	95.85(878)
CCT project				
No	86.76(413)	28.40(135)	87.39 (416)	90.13(429)
Yes	84.62(440)	38.30(199)	98.65(513)	96.92(504)
**Need Factor**				
Self-rated health status				
Low	91.08(60)	25.70(19)	91.89(68)	94.59(70)
Medium	83.04(235)	37.80(107)	92.23(261)	87.99(249)
High	87.32(558)	32.60(208)	93.90(600)	96.09(614)
Health service satisfaction				
Low	76.92(20)	11.50(3)	96.15(25)	92.31(24)
Medium	78.79(104)	31.10(41)	92.42(122)	80.30(106)
High	86.99(729)	34.60(290)	93.32(782)	95.82(803)
**Total**	70.94(288)	33.53(334)	93.27(929)	93.67(933)

### Disparities of maternal service utilization based on socioeconomic characteristics

By calculating risk difference(RD) and relative risk(RR) of maternal service utilization percentage (detailed formulation are listed below), disparities in maternal service utilization exsisted among women with different socioeconomic backgrounds.

$$R1=utilization\;rate\;in\;the\;group\;with\;highest\;socioeconomic\;status$$

$$R2=utilization\;rate\;in\;the\;group\;with\;lowest\;socioeconomic\;status\;(reference\;group)$$

$$RD=R1-R2;\;RR=\frac{R1}{R2}$$

If RD = 0 and RR = 1, there was no gap among different socioeconomic groups; if RD < 0 and RR < 1, the utilization of maternal services was biased towards women with lower socio-economic conditions; if RD > 0 and RR > 1, the utilization of health services is biased towards women with higher socioeconomic conditions.

Result of range analysis (Table 3) showed that the RD of utilizing 5 + ANC, 8 + ANC, ID and 2 + PNC among different education groups were all larger than 0, and its RR were 1.29, 3.18, 1.08 and 1.10, which indicated that women completed high school or above were more likely to utilize maternal services. RD of utilizing 5 + ANC, 8 + ANC, ID and 2 + PNC between different annual income group were all larger than 0, and its RR were 1.10, 1.41, 1.03 and 1.03, which indicated that women with an annual income above the national poverty line were more likely to utilize maternal services. Through horizontal comparison, it was found that the biggest disparity existed in the utilization of 8 + ANC visits. Through vertical comparison, it was found that disparities among different educational groups were larger than those between income groups.

**Table 3:** Disparities of health service utilization based on socioeconomic characteristics

Socio-economic characteristics	5 + ANC	8 + ANC	ID	2 + PNC
Education				
Primary school or below (%)®	73.82	19.60	90.58	89.53
Junior high school (%)	91.96	33.10	93.62	95.27
Senior high school or above (%)	95.29	62.30	97.91	98.43
RD (%)	21.47	42.7	7.33	8.9
RR	1.29	3.18	1.08	1.10
Per capita household income (yuan)				
≤ 4000(%)®	79.71	27.21	91.63	91.86
> 4000(%)	87.92	38.34	94.52	95.05
RD (%)	8.21	11.13	2.89	3.19
RR	1.10	1.41	1.03	1.03

® is the reference category

### Determinants of 5 + ANC utilization

In univariate logistic regression results (Table 4), living in Yunnan or completing middle school or above indicated a higher possibility of 5 + ANC use. Having over 3 children was associated with a reduced chance of 5 + ANC use. As for enabling factors, over 4000 yuan annual income and receiving health education were associated with a higher possibility of 5 + ANC use. Need factors had no significant influence on 5 + ANC use.

**Table 4:** Determinants of 5 + ANC utilization

Background Characteristics	Univariate Analysis				Multivariate Analysis			
	*p*-value	OR	95%CI		*p*-value	OR	95%CI	
**Predisposing Factors**								
Place of residence								
Gansu®								
Yunnan	< 0.001	9.260	5.879	14.584	< 0.001	7.995	4.873	13.116
Age								
≤ 25®								
26–30	0.103	1.535	0.917	2.568	0.008	2.217	1.227	4.004
> 30	0.159	0.725	0.463	1.134	0.548	0.849	0.496	1.451
Education								
Primary school or below®								
Junior high school	< 0.001	4.057	2.671	6.164	< 0.001	2.505	1.546	4.059
Senior high school or above	< 0.001	7.171	3.536	14.543	0.021	2.519	1.148	5.530
Number of children								
1®								
2	0.147	0.648	0.360	1.164	0.655	0.859	0.442	1.672
≥ 3	< 0.001	0.291	0.160	0.531	0.191	0.618	0.301	1.271
**Enabling factors**								
Per capita household income (yuan)								
≤ 4000®								
> 4000	< 0.001	2.232	1.555	3.205	< 0.001	2.115	1.400	3.195
Distance to health facility (km)								
< 2®								
2–4	0.521	0.806	0.417	1.557				
4–6	0.219	0.693	0.386	1.243				
> 6	0.282	1.375	0.770	2.456				
Health education during ANC								
No®								
Yes	< 0.001	5.472	3.365	8.900	< 0.001	2.985	1.666	5.349
CCT project								
No®								
Yes	0.334	0.839	0.587	1.198				
**Need Factors**								
Self-rated health status								
Low®								
Medium	0.692	1.142	0.591	2.209				
High	0.238	1.607	0.859	3.008				
Health service satisfaction								
Low®								
Medium	0.833	1.114	0.409	3.039	0.670	1.275	0.417	3.895
High	0.144	2.006	0.788	5.107	0.916	0.946	0.338	2.648

® is the reference category

Study variables with *p* values smaller than 0.2 in univariate analysis were included in multivariate regression analysis (Table 4). Women living in Yunnan, aged 26–30, and completing junior high school or above had a larger chance of using 5 + ANC use. For enabling factors, over 4000 yuan annual income and receiving health education during ANC were positive determinants of 5 + ANC use.

### Determinants of 8 + ANC utilization

In univariate logistic regression results (Table 5), living in Yunnan or completing middle school or above indicated a higher possibility of 8 + ANC use. Having over 3 children was associated with a reduced chance of 8 + ANC use. As for enabling factors, over 4000 yuan annual income, receiving health education, participating in the CCT project, or living 2–4 km or over 6 km away from a health facility were associated with a higher possibility of 8 + ANC use. Need factors had no significant influence on 8 + ANC use.

**Table 5:** Determinants of 8 + ANC utilization

Background Characteristics	Univariate Analysis				Multivariate Analysis			
	*p*-value	OR	95%CI		*p*-value	OR	95%CI	
**Predisposing Factors**								
Place of residence								
Gansu®								
Yunnan	< 0.001	4.871	3.549	6.685	< 0.001	4.680	3.167	6.914
Age								
≤ 25®								
26–30	0.623	1.094	0.765	1.564				
> 30	0.338	1.184	0.838	1.672				
Education								
Primary school or below®								
Junior high school	< 0.001	2.025	1.465	2.799	0.054	1.439	0.993	2.083
Senior high school or above	< 0.001	6.765	4.597	9.957	< 0.001	3.679	2.343	5.778
Number of children								
1®								
2	0.264	0.824	0.587	1.157	0.735	1.070	0.722	1.586
≥ 3	< 0.001	0.289	0.188	0.446	0.212	0.720	0.430	1.206
**Enabling factors**								
Per capita household income (yuan)								
≤ 4000®								
> 4000	< 0.001	1.663	1.268	2.183	0.002	1.663	1.208	2.289
Distance to health facility (km)								
< 2®								
2–4	0.004	2.360	1.305	4.268	0.716	1.135	0.573	2.248
4–6	0.313	1.337	0.761	2.352	0.420	0.768	0.404	1.460
> 6	< 0.001	4.795	2.875	7.998	< 0.001	3.180	1.784	5.668
Health education during ANC								
No®								
Yes	0.031	1.814	1.055	3.120	0.427	0.763	0.392	1.486
CCT project								
No®								
Yes	0.001	1.566	1.200	2.044	< 0.001	1.981	1.416	2.771
**Need Factor**								
Self-rated health status								
Low®								
Medium	0.054	1.760	0.991	3.125	0.599	0.827	0.408	1.678
High	0.231	1.397	0.808	2.415	0.273	0.685	0.349	1.347
Health service satisfaction								
Low®								
Medium	0.054	3.454	0.981	12.157	0.123	3.235	0.726	14.413
High	0.023	4.057	1.208	13.626	0.208	2.521	0.597	10.640

® is the reference category

Study variables with *p* values smaller than 0.2 in univariate analysis were included in multivariate regression analysis (Table 5). Women living in Yunnan and completing senior high school had a larger chance of using 8 + ANC. For enabling factors, over 4000 yuan annual income, receiving health education, participating in the CCT project, and living over 6 km away from a health facility were positive determinants of 8 + ANC use.

### Determinants of ID utilization

In univariate logistic regression results (Table 6), completed senior school indicated a higher possibility of ID use. As for enabling factors, participating in CCT the project indicated a higher possibility of ID use. Need factors had no significant influence on ID use.

**Table 6:** Determinants of ID utilization

Background Characteristics	Univariate Analysis				Multivariate Analysis			
	*p*-value	OR	95%CI		*p*-value	OR	95%CI	
**Predisposing Factors**								
Place of residence								
Gansu®								
Yunnan	0.062	0.599	0.349	1.026	0.222	0.672	0.354	1.272
Age								
≤ 25®								
26–30	0.067	1.935	0.955	3.922	0.510	1.289	0.606	2.742
> 30	0.921	0.970	0.535	1.760	0.274	0.683	0.345	1.353
Education								
Primary school or below®								
Junior high school	0.111	1.526	0.908	2.565	0.564	1.185	0.666	2.108
Senior high school or above	0.003	4.864	1.705	13.875	0.016	3.936	1.289	12.016
Number of children								
1®								
2	0.194	1.553	0.799	3.019	0.375	1.394	0.669	2.903
≥ 3	0.473	0.779	0.393	1.542	0.862	1.079	0.458	2.542
**Enabling factors**								
Per capita household income (yuan)								
≤ 4000®								
> 4000	0.073	1.577	0.959	2.593	0.056	1.708	0.985	2.961
Distance to health facility (km)								
< 2®								
2–4	0.304	0.561	0.187	1.688	0.204	0.468	0.145	1.509
4–6	0.218	0.531	0.194	1.455	0.314	0.585	0.206	1.663
> 6	0.191	0.527	0.201	1.378	0.260	0.545	0.189	1.568
Health education during ANC								
No®								
Yes	0.453	1.368	0.603	3.101				
CCT project								
No®								
Yes	< 0.001	10.570	4.781	23.370	< 0.001	10.834	4.785	24.532
**Need Factor**								
Self-rated health status								
Low®								
Medium	0.924	1.047	0.408	2.683				
High	0.503	1.357	0.554	3.323				
Health service satisfaction								
Low®								
Medium	0.503	0.488	0.060	3.986				
High	0.571	0.559	0.074	4.198				

® is the reference category

In multivariate regression analysis results (Table 6), the chance of using ID increased significantly when completed senior high school and participated in CCT project.

### Determinants of 2 + PNC utilization

In univariate logistic regression results (Table 7), living in Yunnan or high education level indicated a higher possibility of 2 + PNC use. Having more children was associated with a reduced chance of 2 + PNC use. As for enabling factors, over 4000 yuan annual income, receiving health education, or participating in CCT projects indicated a higher possibility of 2 + PNC use. Need factors had no significant influence on 2 + PNC use.

**Table 7:** Determinants of 2 + PNC utilization

Background Characteristics	Univariate Analysis				Multivariate Analysis			
	*p*-value	OR	95%CI		*p*-value	OR	95%CI	
**Predisposing Factors**								
Place of residence								
Gansu®								
Yunnan	0.001	2.506	1.482	4.237	0.808	1.087	0.555	2.126
Age								
≤ 25®								
26–30	0.808	1.091	0.539	2.207				
> 30	0.702	0.879	0.455	1.700				
Education								
Primary school or below®								
Junior high school	0.003	2.357	1.352	4.108	0.432	1.300	0.676	2.499
Senior high school or above	0.001	7.329	2.237	24.012	0.030	4.962	1.163	21.165
Number of children								
1®								
2	0.037	0.281	0.085	0.929	0.050	0.259	0.067	0.999
≥ 3	0.001	0.133	0.040	0.443	0.063	0.270	0.068	1.075
**Enabling factors**								
Per capita household income (yuan)								
≤ 4000®								
> 4000	0.059	1.703	1.019	2.846	0.054	1.830	0.991	3.381
Distance to health facility (km)								
< 2®								
2–4	0.160	0.513	0.202	1.302	0.510	0.702	0.245	2.009
4–6	0.058	0.440	0.188	1.029	0.086	0.448	0.179	1.121
> 6	0.057	2.607	0.972	6.994	0.119	2.318	0.807	6.662
Health education during ANC								
No®								
Yes	< 0.001	10.502	5.918	18.637	< 0.001	6.197	2.881	13.328
CCT project								
No®								
Yes	< 0.001	3.451	1.929	6.174	0.003	2.689	1.415	5.111
**Need Factor**								
Self-rated health status								
Low®								
Medium	0.110	0.418	0.144	1.219	0.017	0.212	0.059	0.757
High	0.540	1.403	0.475	4.149	0.170	0.404	0.110	1.477
Health service satisfaction								
Low®								
Medium	0.160	0.340	0.075	1.530	0.668	0.675	0.112	4.069
High	0.391	1.912	0.434	8.413	0.654	1.496	0.258	8.676

® is the reference category

Study variables with a *p*-value smaller than 0.2 in univariate analysis were included in multivariate regression analysis (Table 7). Women with 2 children had a reduced chance of 2 + PNC utilization. For enabling factors, health education and CCT project participation were positively associated with the chance of 2 + PNC use. As for need factor, women with a medium-level self-rated health status were more likely to use 2 + PNC.

## Discussions

The present study assessed the utilization and determinants of maternal health services among women residing in poverty-stricken rural areas in China. The utilization rates of ID and 2 + PNC services were both above 90%, but the 8 + ANC utilization rate was only 33%. The gap in utilization rates of different maternal services is because of the BPHS. BPHS provides free ANC, ID, and 2 + PNC services to women, but it only covered 5 ANC visits instead of 8 visits. Besides, disparities in utilization of maternal services among different socioeconomic groups existed and women with higher educational background and annual income tend to use more health services, which was in line with the results of regression analysis.

### Predisposing factors

Place of residence is an important influencing factor in maternal health service utilization. Women in Yunnan are more likely to use 5 + ANC and 8 + ANC. The availability of health facilities and health professionals is directly associated with the utilization of maternal services [38]. Compared with Gansu province, Yunnan has better economic development and more health facilities that can provide quality care services, which leads to more ANC services use. In 2020, Yunnan had 147 maternal and childcare service centers whereas Gansu only had 99 [14]. 36,466 country doctors worked in Yunnan whereas only 18,088 worked in Gansu [14].

Level of education, which is often positively associated with one’s health literacy, is a critical determinant of maternal services use [39–41]. Our findings indicate that the higher the education level, the higher the probability of using ANC and ID, which is in line with recent studies in Eswatini, India, and Ethiopia [42–44]. Multiple parturitions have an adverse effect on maternal service utilization since experienced pregnant women tend to ignore the importance of maternal services, which is in line with studies by Zhu and Wang [45, 46].

### Enabling factors

Financial factors such as CCT participation and annual income outcompete other enabling factors in influencing maternal services use. Participation in CCT is the most influential enabling factor that shows positive associations with all 3 maternal services utilization. Providing financial incentives to poor women with preset conditions not only promotes women’s motivation of using maternal services but also alleviated their financial burden of purchasing services. CCT has also been proven to be effective in improving health outcomes and encouraging health service utilization in India, Brazil, Mexico, etc. [47–51]. Annual income is another determinant of 8 + ANC utilization. Since not all 8 ANC visits are covered by health insurance, women with relatively high per capita income tend to use more ANC services. Out-of-pocket fees hinder the utilization of maternal services, which is in line with findings from the Maternal Immunisation and Antenatal Care Situational analysis (MIACSA) project in low and middle-income countries [52].

Besides financial factors, health education is another influencing factor in maternal service utilization. Receiving health education during ANC has a positive association with the chance of 2 + PNC use since health education may improve one’s health literacy and have an indirect influence on one’s healthy behavior. This is in line with previous studies in South Asia showing that women’s education and exposure to health information were positively and significantly correlated with their maternal health service utilization [53, 54]. Especially in areas where the average education level of women is relatively low, receiving health education makes a huge difference in their health choice and behavior. What’s more, distance to health facilities was found to be a negative influencer of health service utilization in many studies since large indirect cost (traffic, lost income, time expense, etc.) ruins people’s motivation to pursue health services [55, 56]. However, it is not the case in the present study. Women living over 6 km away from health facilities had higher possibilities of using 8 + ANC. A possible explanation of this result is that there was a positive correlation between respondents’ annual income and living distance, and annual income is positively associated with maternal service utilization.

### Need factors

Some previous studies reported need factors as strong determinants of health service utilization [57, 58], but it is not the case in the present study. Regarding maternal health service utilization among poor women, only “self-rated health status” has a significant association with 2 + PNC use. The higher the self-rated health, the smaller the chance of using 2 + PNC. One possible explanation is that postnatal care visits often took place at home instead of the hospital, doctors normally provide home visit services to women, which may make women unerestimate its importance. Besides, because of the “sitting-month” custom, women may not welcome visitors after childbirth, which may harm the demand of PNC services and women with high self-rated health score may be less likely to seek PNC services. Women’s satisfaction with health services shows little association with maternal service use, which is in line with a study conducted in Georgia reporting that high satisfaction doesn’t ensure efficient use of maternal resources [59].

### Recommendations for maternal service utilization improvement

Based on the results of the present study, we come up with the following recommendations. First, providing health education to women is critical in improving health service utilization. Disparity existed among different education group. Lacking knowledge about pregnancy and childbirth leads to underutilization of maternal health services since women are not able to realize the importance of maternal services. Poverty often connects to low education levels, which makes health education even more important since women’s basic health literacy is relative low with little perspective about the severity of not using maternal resources efficiently. Besides, providing health education will make an indirect influence on women’s self-rated health status by helping women build correct awareness of their health status. That way they will be able to consume adequate health services they need. disparities in maternal services utilization happened among different socioeconomic groups. Second, the government should initiate financial incentive programs focusing on maternal service use such as CCT. There was a maternal service utilization gap between women under poverty line and above the poverty line. Projects like CCT that can target poor household and provide financial incentives with conditions can help reduce the economic burden caused by using maternal services can encourage more health utilization behavior by women. At the same time, awarding money to healthy behavior indirectly alleviates the target population’s poverty.

### Limitations of the study

The present study has several limitations. First, the study was conducted in poverty-stricken rural areas in two provinces of China. Therefore, the results may not be generalized to the general population or economically developed areas. Second, the present study only identifies influencing factors of maternal service utilization but is not able to weigh each determinant’s contribution to maternal service utilization, which is the major limitation of the study. Third, the present study only analyzes the association of different factors and maternal service utilization but does not link service utilization to maternal health outcomes. Further studies can be done on accessing the association between maternal service utilization and health outcomes.

## Conclusions

This study shows a strong association between both predisposing and enabling factors and maternal services utilization. Predisposing factors such as place of residence, age and education level, enabling factors such as annual income, and health education contribute to 5 + ANC visits; predisposing factors such as place of residence, and education level along with enabling factors such as CCT participation, annual income, and distance to health facilities contribute to more than 8 + ANC visit; predisposing factors such as place of residence, and education level along with enabling factors CCT participation contribute to ID; predisposing factors number of children, enabling factors CCT participation and health education along with need factor self-rated health status contribute to 2 + PNC visits.

We conclude that in order to improve maternal health service utilization and eventually improve maternal health status to meet SDG and healthy China 2030’s goal, more health education should be provided and financial incentive projects targeting pregnant women with low household income should be implemented.

## Data Availability

The datasets used and analyzed during the current study are available from the corresponding author on reasonable request.

## Abbreviations

8 + ANC: At least 8 antenatal care visits
ID: Institutional delivery
2 + PNC: At least 2 postnatal care
